# WTAP/IGF2BP3-mediated GBE1 expression accelerates the proliferation and enhances stemness in pancreatic cancer cells via upregulating c-Myc

**DOI:** 10.1186/s11658-024-00611-8

**Published:** 2024-07-03

**Authors:** Weiwei Jin, Yanru Yao, Yuhan Fu, Xiangxiang Lei, Wen Fu, Qiliang Lu, Xiangmin Tong, Qiuran Xu, Wei Su, Xiaoge Hu

**Affiliations:** 1 Zhejiang Key Laboratory of Tumor Molecular Diagnosis and Individualized Medicine, Zhejiang Provincial People’s Hospital, Affiliated People’s Hospital, Hangzhou Medical College, Hangzhou, China; 2 General Surgery, Cancer Center, Department of Gastrointestinal and Pancreatic Surgery, Zhejiang Provincial People’s Hospital (Affiliated People’s Hospital), Hangzhou Medical College, Hangzhou, Zhejiang China; 3 Hangzhou Medical College, Hangzhou, China; 4 The Medical College of Qingdao University, Qingdao, China; 5 Department of Hepatobiliary and Pancreatic Surgery, The First Affiliated Hospital, Zhejiang University School of Medicine, Hangzhou, China; 6 Zhejiang Provincial Key Laboratory of Pancreatic Disease, Hangzhou, China

**Keywords:** Pancreatic cancer, GBE1, m6A modification, IGF2BP3, WTAP, Proliferation

## Abstract

**Background:**

Pancreatic cancer (PC) is one of the most malignant cancers with highly aggressiveness and poor prognosis. N6-methyladenosine (m6A) have been indicated to be involved in PC development. Glucan Branching Enzyme 1 (GBE1) is mainly involved in cell glycogen metabolism. However, the function of GBE1 and Whether GBE1 occurs m6A modification in PC progression remains to be illustrated.

**Methods:**

The clinical prognosis of GBE1 was analyzed through online platform. The expression of GBE1 was obtained from online platform and then verified in normal and PC cell lines. Lentivirus was used to generated GBE1 stable-overexpression or knockdown PC cells. Cell Counting Kit (CCK-8), colony formation assay, sphere formation assay and flow cytometry assay were conducted to analyze cell proliferation and stemness ability in vitro. Subcutaneous and orthotopic mouse models were used to verify the function of GBE1 in vivo. RNA immunoprecipitation (RIP) assay, RNA stability experiment and western blots were conducted to explore the molecular regulation of GBE1 in PC.

**Results:**

GBE1 was significantly upregulated in PC and associated with poor prognosis of PC patients. Functionally, GBE1 overexpression facilitated PC cell proliferation and stemness-like properties, while knockdown of GBE1 attenuated the malignancy of PC cells. Importantly, we found the m6A modification of GBE1 RNA, and WTAP and IGF2BP3 was revealed as the m6A regulators to increase GBE1 mRNA stability and expression. Furthermore, c-Myc was discovered as a downstream gene of GBE1 and functional rescue experiments showed that overexpression of c-Myc could rescue GBE1 knockdown-induced PC cell growth inhibition.

**Conclusions:**

Our study uncovered the oncogenic role of GBE1/c-Myc axis in PC progression and revealed WTAP/IGF2BP3-mediated m6A modification of GBE1, which highlight the potential application of GBE1 in the targeted therapy of PC.

**Supplementary Information:**

The online version contains supplementary material available at 10.1186/s11658-024-00611-8.

## Background

According to the GLOBOCAN 2020 from International Agency for Research on Cancer (IARC), pancreatic cancer (PC) has been reported to be the seventh leading causes of cancer-associated deaths worldwide, with a five-year survival around 10% [1, 2]. Due to the lack of specific symptoms and early-diagnostic biomarkers, up to 80% patients were diagnosed with PC at an advanced stage. The five-year survival of the patients who get a surgical resection is only 15–25% [3]. Despite the improvements in PC clinical treatment in recent years, the mortality of PC remains high, and the number of deaths and new cases in 2020 is almost the same [1]. Therefore, it is valuable to uncover the molecular mechanism of PC development, and then explore the novel targeted therapy for PC.

Hundreds of RNA modifications have been identified in RNAs, such as N1-methyladenosine (m1A), N6-methyladenosine (m6A), 5-methylcytosine (m5C) and 7-methylguanosine (m7G), etc. [4]. Among these modifications, RNA m6A modification was defined as the most abundant RNA modification in eukaryotes, which has attracted much attention since it was firstly identified in 1974 [5, 6]. Reported as a dynamic and reversible process, m6A modification is triggered by methyltransferase proteins (also called “writers”, including METTL3, METTL14, WTAP, KIAA1429, ZC3H13 and RBM15), and then removed by demethylase proteins (also called “erasers”, including FTO [7] and ALKBH5 [8]). Additionally, m6A modification is recognized by m6A binding proteins (also called “readers”, including IGF2BPs, YTHDFs and YTHDCs), thus affecting RNA fate [9, 10]. As reported, RNA m6A modification was present on the 5’UTR, CDS, 3’UTR of both protein coding RNAs (mRNAs) and non-coding RNAs (ncRNAs). Numerous studies have demonstrated that RNA m6A modification plays crucial roles in the malignant progression of human cancers including PC [11, 12]. At present, IGF2BP2 [13], YTHDF2 [14–18], YTHDC1 [19], NKAP [20] and CNBP [21] have been identified as m6A readers to regulate the maturation, alternative splicing, stability, translation or degradation of RNAs in PC.

At present, m6A modifications of coding and non-coding RNAs in PC have been revealed, which contributed to the malignant progression of PC. As reported, m6A levels was significantly upregulated in PC tissues when compared to normal tissues [15]. METTL14 inhibited PERP expression in an YTHDF2-m6A-dependent manner, enhancing tumor growth and metastasis of PC cells [15]. Lin et al. revealed that FTO enhanced NEDD4 expression via YTHDF2-m6A-dependent manner, forming an oncogenic FTO/NEDD4 axis to trigger gemcitabine resistance of PC cells through modulating PTEN/PI3K/AKT [22]. In addition to coding RNAs, m6A modification of non-coding RNAs were also uncovered. Zhang et al. found that DDIT4-AS1 was highly expressed in PC and predicted poor prognosis of PC patients, which was suppressed by ALKBH5 via m6A-dependent way. DDIT4-AS1 enhanced the stemness and chemoresistance of PDAC cells through enhancing mTOR pathway [23]. We previously reported that IGF2BP2, serving as an m6A reader, could bind to and stabilize DANCR, therefore promoting the cell growth and stemness of PC [13].

Glucan Branching Enzyme 1 (GBE1), mainly distributed in liver and muscle tissue, is a glycogen branching enzyme and involved in the transportation of glucosyl to glycogen chain, which is essential for glycogen accumulation and its solubility upregulation [24, 25]. Whether m6A modification was present on GBE1 mRNA has not been elucidated. GBE1 mutations have been reported to be associated with adult polyglucosan body disease (APBD) and Glycogen Storage Disease Type IV (GSD IV) [26, 27]. Recently, GBE1 was reported as an oncogene in the cell growth, migration, stemness, glycolysis and anti-tumor immunity of lung adenocarcinoma (LUAD) [28, 29]. However, the role of GBE1 in PC has not been reported. C-Myc is a well-known oncogenic molecular, which is often upregulated in various human cancers and participates in modulating cell fate including cell growth, chemoresistance and metabolism [30]. The regulation mechanism of GBE1 and c-Myc needs to be further demonstrated.

In this study, we revealed the function and molecular mechanism of GBE1 in PC carcinogenesis. We found that GBE1 overexpression promoted PC cell growth, stemness-like properties, while loss of GBE1 suppressed the malignant progression of PC. Furthermore, c-Myc was uncovered as a downstream target of GBE1 in regulating PC cell growth. Importantly, we firstly revealed the m6A modification of GBE1 mRNA, and WTAP and IGF2BP3, acting as a m6A regulators, could bind to and stabilize GBE1 mRNA through m6A modification. Overall, our study demonstrated the oncogenic role of GBE1/c-Myc axis in PC and WTAP/IGF2BP3-mediated upregulation of GBE1 via m6A modification, which providing a promising therapeutic target for PC treatment.

## Methods

### Cell Culture and transfection

BXPC3 (Serial: TCHu 12) and SW1990 (Serial: TCHu201) were purchased from Cell Bank of the Chinese Academy of Sciences (Shanghai, China). BXPC3 was growth in RPMI 1640 medium (Hyclone) supplied with 10% FBS (BI) and 1% Penicillin–Streptomycin Solution (Solarbio). SW1990 was growth in high-glucose DMEM (Hyclone) medium containing 10% FBS (BI) and 1% Penicillin–Streptomycin Solution (Solarbio). BXPC3 and SW1990 was cultured in cell incubator (Thermo Fisher Scientific) with 5% CO2 at 37 ℃. Lipofectamine 3000 (Thermo Fisher Scientific) was used for plasmid and siRNA transfections according to the manufacturer’s instructions.

### Plasmids construction and siRNA

3 × Flag-tagged GBE1-overexpression (OE) virus and negative control (NC) virus were purchased from Hanbio Biotechnology. GBE1-knockdown (sh-GBE1) virus and its negative control (sh-NC) virus were purchased from Hanbio Biotechnology Co. Ltd. as well. Stable cell lines were obtained through puromycin selection. IGF2BP3 siRNA, GBE1 siRNA, c-Myc siRNA and negative control siRNA were purchased from GenePharma.

### Western blot

Cells were harvested and lysed for 1 h at 4 ℃. Then, the cell lysate was centrifuged at 12,000 rpm for 20 min at 4 ℃, and the supernatant was collected, which was followed by protein concentration via BCA method. Proteins were separated by SDS–PAGE and then transferred to PVDF membrane. Then, the membrane was blocked with 5% skim milk for 1 h at room temperature. The primary antibody was incubated with membrane at 4℃ overnight. The membrane was washed 3 times for 10 min with TBST, followed by incubating with secondary antibody for 1 h and then washed 3 times for 10 min with TBST. The protein was detected by ChemiDoc™ MP Imaging System (Bio-Rad). The reagents used in this study were as follows: GBE1 (Proteintech, Cat#14642-1-AP), IGF2BP3 (Proteintech, Cat#14642-1-AP), WTAP (Proteintech, Cat#10200-1-AP), c-Myc (Abcam, ab32072), GAPDH (Beyotime, AG019), Actin (Beyotime, AA128), 3-Deazaadenosine/DAA (MedChemExpress, HY-W013332).

### RNA extraction and quantitative real-time PCR

The total RNA of cells was extracted by RNAiso Plus (Takara) according to the manufacturer’s instructions. Then, cDNA was generated from total RNA through PrimeScript™ RT reagent Kit (Takara). Quantitative real-time PCR was carried out by SYBR Green (Bio-Rad) in Applied Biosystems® 7500 Real-Time PCR Systems (Thermo Fisher Scientific). Finally, the data were analyzed. Primers used in this study were purchased from Tsingke Biotechnology and were listed as below. GBE1 (Forward-AGACTCCTGGAGATCGACCC, Reverse-ATCAGCACATCTGTGGACGC), IGF2BP3 (Forward-CCATGTGATTTGCCTCTGCG, Reverse-ACTGGGTCTGTTTGGTGATGT), WTAP (Forward- GGGAAAGGACGGGGAGTGTT, Reverse-TTGGTCATCTTGAATCAGGAGAGT), c-Myc (Forward-TGCTCCATGAGGAGACACC, Reverse-CTTTTCCACAGAAACAACATCG), GAPDH (Forward-GTCTCCTCTGACTTCAACAGCG, Reverse-ACCACCCTGTTGCTGTAGCCAA).

### CCK-8 assay

Cell proliferation was analyzed by Cell Counting Kit (CCK-8, Yeasen). BXPC3 and SW1990 cells was seeded in 96 well plate in a density of 3500 cells/well. 10% volume CCK-8 was added to the cell medium of 96 well plate at 24 h, 48 h and 72 h separately. After incubation with CCK-8 for 0.5 ~ 4 h, OD450 value of pancreatic cancer cells was tested. Finally, the pancreatic cell growth curve was analyzed based on the OD450 value.

### Colony formation assay

Cell proliferation ability was also measured by colony formation assay. Briefly, BXPC3 and SW1990 cells were harvested as single cell suspension and subsequently seeded into 6-well plate at a density of 500 cells/well. When the single cell colony was grown to the proper size, cells were fixed in 4% paraformaldehyde solution (Sangon Biotech) in PBS for 30 min at room temperature. After washing with 1 × PBS for three times, cells were stained with crystal violet solution (Yeasen) for 30 min followed by washing with 1 × PBS for three times. Every 6-well plate was taken pictures. The relative colony formation rate was calculated as below: colony number of experimental group/colony number of negative control group × 100%.

### Sphere formation assay

Cells were seeded into ultralow attachment 6-well plate at a density of 50,000 cells/well, which were cultured in DME/F12 medium supplemented with N2 (Thermo Fisher Scientific), 10 ng/ml human epidermal growth factor (EGF) and human basic fibroblast growth factor (bFGF). 1 ml medium was added every three days. After cultured for 10 days, cell spheres were taken pictures by microscope (Nikon). Each experiment was repeated for three times.

### Flow cytometry assay

A total of 1 × 10^6^ BXPC3 or SW1990 cells were harvested, and then washed with 1 × PBS for three times via centrifuging at 1000 rpm for 5 min. Then, the washed cells were resuspended to 100ul 1 × PBS, and PE-CD24 (eBioscience, Cat #12–0247-42), FITC-CD44 (eBioscience, Cat# 11-0441-82) or APC-CD133 (eBioscience, Cat#17–1338-42) antibody (5ul/test) were added. After incubation with antibody for 30 min at room temperature without light. Cells were washed with 1 × PBS for three times via centrifuging at 1000 rpm for 5 min and finally subjected to analysis by flow cytometry (Beckman). The relative cell population of CD24^+^, CD44^+^ and CD133^+^ cells were calculated as normalized to control group.

### RNA immunoprecipitation

In this study, IGF2BP3 (Proteintech, Cat#14642-1-AP) antibody were used to precipitate GBE1 RNA and m6A antibody (Sigma, MABE1006) was used to precipitate m6A-modified RNAs. For IGF2BP3 RIP, we used EZ-Magna RIP™ RNA-Binding Protein Immunoprecipitation Kit (Millipore, Cat#17-701) according to the manufacturer’s instructions. Briefly, cells were lysate by RIP lysis buffer supplemented with protein inhibitor cocktail and RNase inhibitor. After IGF2BP3 antibody or negative control IgG were incubated with magnetic beads, supernatant of RIP cell lysate was added to the antibody-magnetic beads complex to obtain protein-antibody-magnetic beads complex and then purify RNA according to the manufacturer’s instructions. Finally, RT-qPCR was performed to analyzed the target RNA enrichment.

As referred to m6A RIP, EZ-Magna RIP™ RNA-Binding Protein Immunoprecipitation Kit was used as described in IGF2BP3 RIP to analyze the m6A enrichment of target RNA.

### Animal experiment

Female nude mice (4–6 weeks old) were purchased from the SLAC Laboratory Animal Co., Ltd (Shanghai, China), and the animal protocol was approved by the Animal Ethical and Welfare Committee of Zhejiang Provincial People’s Hospital in accordance with the Basel Declaration. Nude mice were then divided into different groups (*n* = 5 per group) randomly. For NC and GBE1 groups, 1 × 10^6^ BXPC3 cells/150ul solution (RPMI1640:Matrixgel = 1:1) were subcutaneously injected into the back of mice. For sh-NC and sh-GBE1 groups, 5 × 10^5^ BXPC3 cells/150ul solution (RPMI1640:Matrixgel = 1:1) were subcutaneously injected into the back of mice. Mouse weight and tumor volume were measured every 5 days in each group. Mice were sacrificed after 20 days. Curve of mice weight and tumor volume were analyzed (*n* = 5). Tumor volume was calculated as (length × width^2^)/2.

For pancreatic cancer orthotopic mouse model, mice were anesthetized by sodium pentobarbital. 3 × 10^6^ BXPC3 cells in 50ul RPMI 1640 medium were injected into the pancreas of nude mice with insulin syringe (*n* = 5 per group). After the peritoneum and skin were closed separately, the mice were placed on a heating plate for their waking up. The mouse weight was measured every 5 days. One month later, the mice were all sacrificed.

### Immunohistochemistry (IHC)

The nude mice tumor tissues were fixed and then subjected to paraffin-embedded. Immunohistochemistry staining was conducted as previously described [13]. Antibodies including GBE1 (Origene, TA500801), c-Myc (Abcam, ab32072) and Ki-67 (CST, 12202) were used for IHC staining. Image J was used to analyzed the IHC staining of GBE1, c-Myc and Ki67, which was shown as integrated optical density (IOD) value of area.

### Bioinformatic analysis

The Gene Expression Profiling Interactive Analysis (GEPIA) database (http://gepia.cancer-pku.cn/) is a freely available platform based on TCGA and GTEx data [31]. The expression of GBE1 in PC and normal tissues was analyzed by GEPIA. The expression relationship between GBE1 and WTAP or IGF2BP3 was analyzed by Correlation Analysis in GEPIA database. 0.05 was used as *p*-value cutoff.

XIANTAO platform (https://www.xiantaozi.com/) is an online bioinformatics analysis web tool. The expression of GBE1 in PC and normal tissues was analyzed via XIANTAO platform (Mann–Whitney *U* test). We used Log2(TPM + 1) for log-scale in *Y* axis. The prognosis (Overall Survival, Disease Specific Survival and Progress Fee Survival) of GBE1 expression in PC was analyzed by XIANTAO platform. The Kaplan–Meier (KM) plotter database (https://kmplot.com/analysis/) is an online survival analysis tool. The association between GBE1 expression and relapse free survival rate of PC patients were analyzed by KM plotter.

### Statistical analysis

Data were shown as mean ± Standard Deviation (SD). All statistical analysis were performed by GraphPad Prism software. The student t test was used to compare the variable between two groups. Multiple groups were analyzed by ANOVA test. *p* < 0.05 was considered as significant.

## Results

### Upregulation of GBE1 predicted poor prognosis in PC patients

**Fig. 1:**
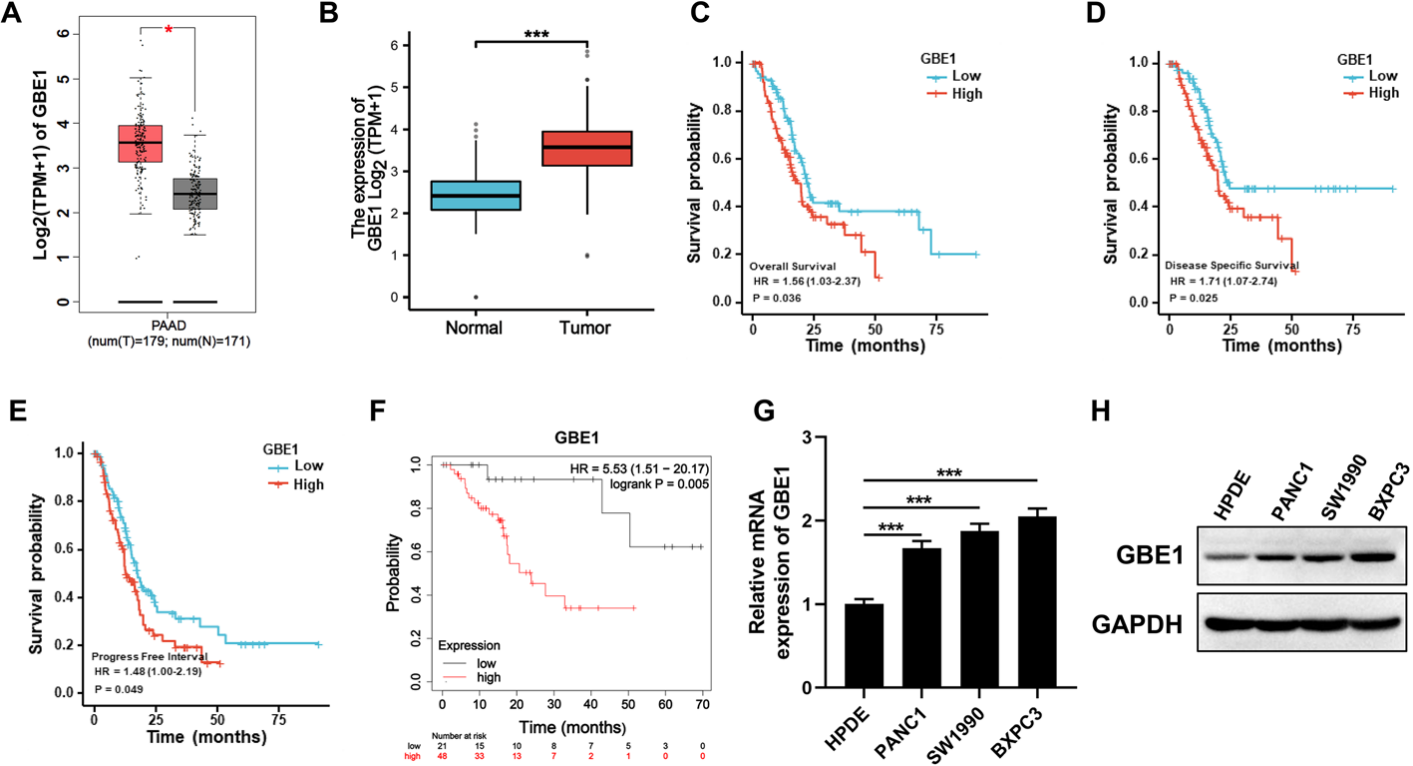
GBE1 was highly expressed in PC and predicted poor prognosis of PC patients.** A** GBE1 expression in PC and normal tissues was analyzed by GEPIA platform. We used Log_2_(TPM + 1) for log-scale in Y axis. 0.05 was used as p-value cutoff. **B** XIANTAO platform was used to explore the GBE1 expression in PC and normal tissues. *p* < 0.001. **C** Overall survival (OS) of PC patients was analyzed on XIANTAO platform. **D** Disease specific survival (DSS) of PC patients was analyzed on XIANTAO platform. **E** Progression free interval (PFI) of PC patients was analyzed on XIANTAO platform. **F** Relapse free survival (RFS) of PC patients was analyzed on Kaplan–Meier plotter platform. **G** RNA expression of GBE1 in PC cells and normal HPDE cells (one-way ANOVA test). **H** Protein expression of GBE1 in PC cells and normal HPDE cells. ***p < 0.0001

To explore GBE1 expression in PC, we firstly analyzed its expression via public bioinformatics platforms. As revealed by Gene Expression Profiling Interactive Analysis (GEPIA) platform and XIANTAO platform (https://www.xiantaozi.com/) based on RNA-sequencing datasets, GBE1 was significantly highly expressed in PC tissues when compared with adjacent normal tissues (Fig. 1A, B). Moreover, analysis of PC RNA-sequencing through XIANTAO platform further showed that high expression of GBE1 was significantly associated with poor overall survival (OS) (Fig. 1C), disease specific survival (DSS) (Fig. 1D) as well as progress free interval (PFI) (Fig. 1E) in PC patients. Consistent with above results, analysis of PC RNA-sequencing through Kaplan–Meier plotter platform (https://kmplot.com/analysis/) also showed that upregulation of GBE1 predicted poor relapse free survival (RFS) in PC patients (Fig. 1F). Then, we verified GBE1 expression in PC cells. As shown in Fig. 1G, H and Figure S1A, higher expression of GBE1 was also observed in both RNA and protein level of PC cells when compared to normal human pancreatic duct epithelial (HPDE) cells. Taken together, GBE1 was overexpressed in PC and predicted poor prognosis of PC patients.

### GBE1 overexpression enhanced cell growth and stemness-like properties of PC cells

**Fig. 2:**
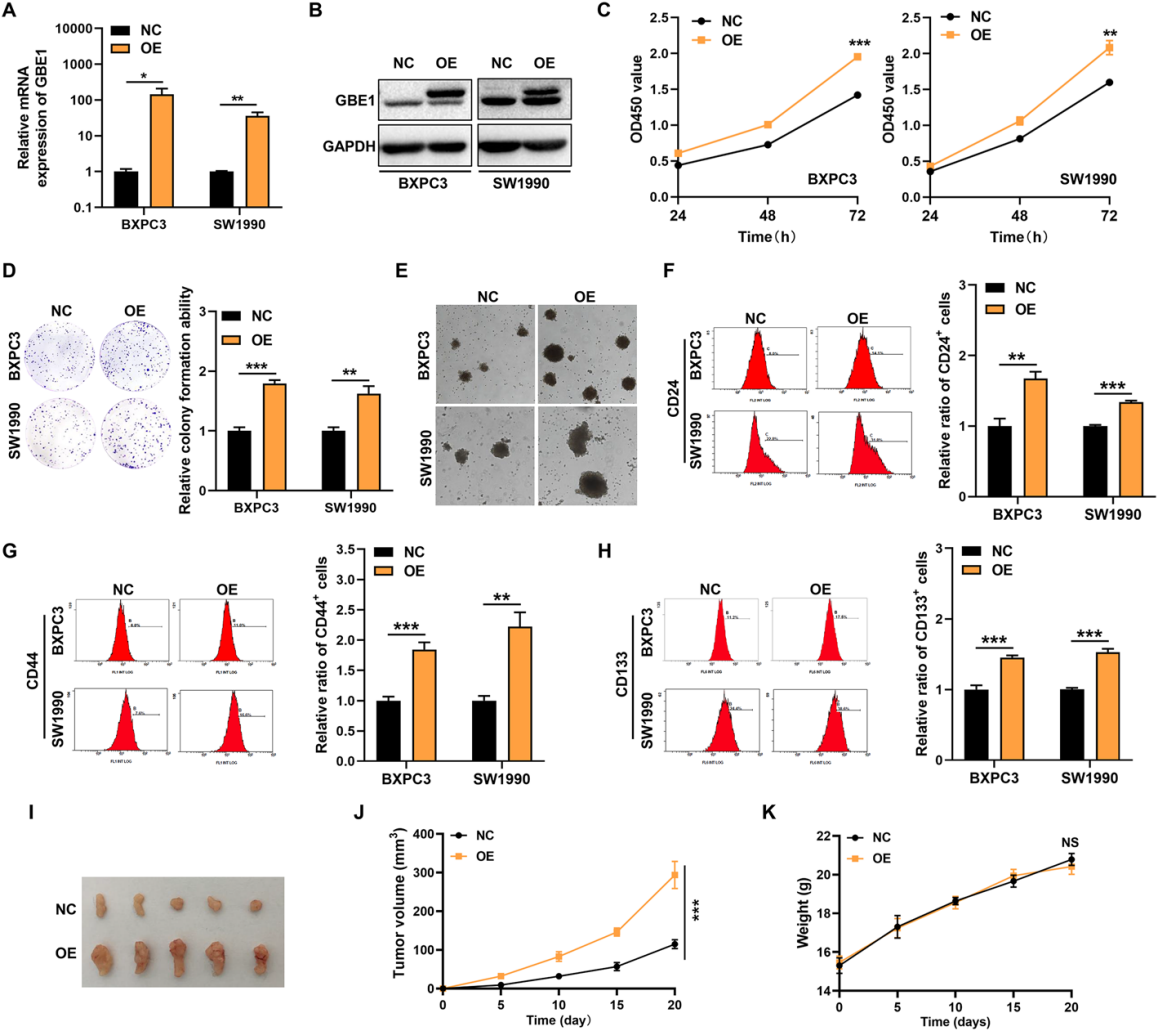
GBE1 overexpression promoted the growth and stemness-like properties of PC cells. **A** RT-qPCR analysis of GBE1 overexpression efficiency in PC cells (*t* test). **B** Western blot analysis of GBE1 overexpression efficiency in PC cells. **C** GBE1 increased PC cell proliferation revealed by CCK8 assay (*t* test). **D** GBE1 enhanced colony formation ability of PC cells, and colony statistical analysis was shown on the right (t test). **E** GBE1 promoted sphere formation ability of PC cells. GBE1 promoted the percentage of CD24^+^ (**F**), CD44^+^ (**G**) and CD133^+^ (**H**) population of PC cells revealed by flow cytometry. The statistical results of flow cytometry were showed on the right (*t* test). **I** GBE1 overexpression promoted tumor growth of PC cells in vivo as compared to negative control (NC) cells. NC or GBE1 BXPC3 cells (1 × 10^6^ cells/150ul, *N* = 5) were subcutaneously injected into the back of nude mice. Twenty days later, tumors were removed from mice. Tumor volume (**J**) and mice weight (**K**) of both NC and GBE1 groups were measured and analyzed every 5 days (*t* test). *p < 0.05, **p < 0.01, ***p < 0.001. *NS* No significant

To explore the function of GBE1 in PC, we generated GBE1 stable expression BXPC3 and SW1990 cell lines. The overexpression efficiency of GBE1 was analyzed in both RNA (Fig. 2A) and protein level (Fig. 2B, Fig S1B). The CCK8 assay showed that GBE1 overexpression enhanced PC cell proliferation (Fig. 2C). The colony formation assay further revealed the role of GBE1 to promote PC cell growth (Fig. 2D).

Cancer stem cells (CSCs) are a subset of cells, characterized by the ability of self-renew and tumorigenesis. In order to determine the role of GBE1 in stemness-like properties of PC cells, we performed the sphere formation assay which is a typical feature of CSCs [32, 33]. Overexpression of GBE1 significantly enhanced sphere formation ability of BXPC3 and SW1990 cells, since a larger size of spheres were observed in GBE1-overexpressed cells when compared with negative control cells (Fig. 2E). As previously demonstrated, CD24, CD44 and CD133 were cell surface markers of PC CSCs [34, 35]. Consistent with the above results, a significant increase of CD24^+^, CD44^+^ and CD133^+^ cell population were observed in GBE1-overexpressed BXPC3 and SW1990 cells (Fig. 2F–H). Overall, GBE1 overexpression enhanced PC cell proliferation and stemness-like properties in vitro. In order to explore the effect of GBE1 in PC cell growth in vivo, we subcutaneously injected PC cells into the back of nude mice. GBE1 overexpression dramatically enhanced tumor growth since the tumor size was larger in GBE1 overexpression group when compared to negative control group (Fg. 2I, J, Fig. S2A), and the body weight changes of nude mice in each group were shown in Fig. 2K. Taken together, GBE1 enhanced cell growth both in vitro and in vivo.

### Knockdown of GBE1 suppressed cell growth and stemness-like properties of PC cells

**Fig. 3:**
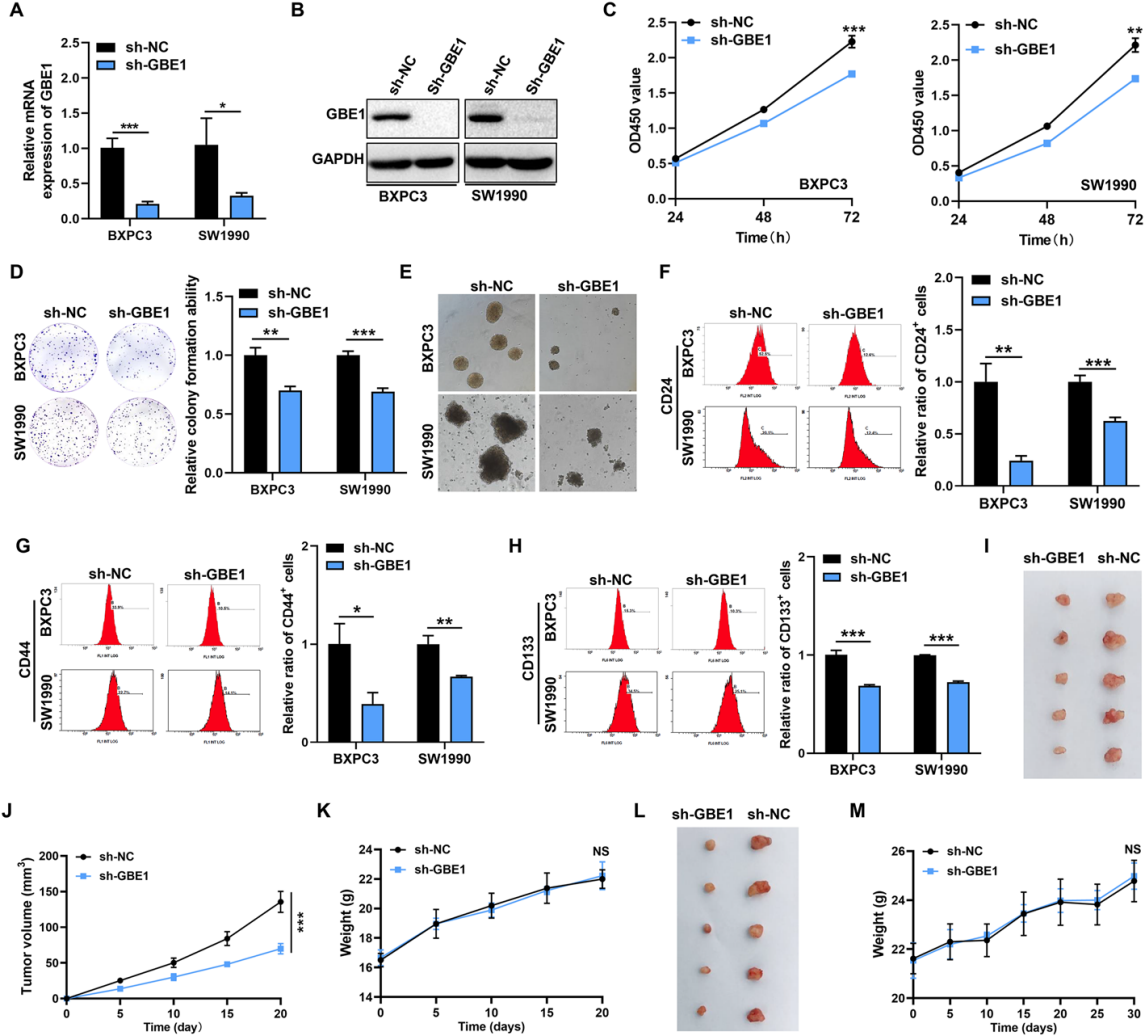
GBE1 deficiency suppressed the PC cell growth and stemness-like properties. **A** RT-qPCR analysis of GBE1 knockdown efficiency in PC cells. t test. **B** Western blot analysis of GBE1 knockdown efficiency in PC cells. **C** GBE1 knockdown suppressed PC cell proliferation revealed by CCK8 assay (*t* test). **D** GBE1 knockdown reduced colony formation ability of PC cells, and colony statistical analysis was shown on the right (t test). **E** GBE1 knockdown suppressed sphere formation ability of PC cells. GBE1 knockdown decreased the percentage of CD24^+^ (**F**), CD44^+^ (**G**) and CD133^+^ (**H**) population of PC cells revealed by flow cytometry. The statistical results of flow cytometry were showed on the right (t test). **I** GBE1 knockdown suppressed tumor growth of PC cells in vivo when compared to negative control (sh-NC) cells. sh-NC or sh-GBE1 BXPC3 cells (5 × 10^5^ cells/150ul) were subcutaneously injected into the back of nude mice. Twenty days later, tumors were removed from mice. Tumor volume (**J**) and mice weight (**K**) of both sh-NC and sh-GBE1 groups were measured and analyzed every 5 days (*t* test). **L** GBE1 knockdown suppressed tumor growth of PC cell as determined by orthotopic mouse model. sh-NC or sh-KD BXPC3 cells (3 × 10^6^ cells/50ul, *N* = 5) were orthotopically injected into the pancreas of nude mice. One month later, tumors were removed from mice. Mice weight (**M**) of both sh-NC and sh-GBE1 groups were measured and analyzed every 5 days (*t* test). *p < 0.05, **p < 0.01, ***p < 0.001. *NS* No significant

To verify the role of GBE1 in PC cell proliferation and stemness-like properties, we generated GBE1 stable knockdown BXPC3 and SW1990 cells. The knockdown efficiency of GBE1 was showed in both RNA (Fig. 3A) and protein levels (Fig. 3B, Fig S1C). Contrary to GBE1 overexpression, loss of GBE1 suppressed PC cell proliferation (Fig. 3C) as well as colony formation ability (Fig. 3D). Furthermore, knockdown of GBE1 decreased cell sphere formation ability of both BXPC3 and SW1990 cells, as the sphere size was smaller when GBE1 was knockdown (Fig. 3E). Moreover, an obvious decrease of CD24^+^, CD44^+^ and CD133^+^ cell population was revealed upon knockdown of GBE1 in both BXPC3 and SW1990 cells (Fig. 3F–H). To verify the function of GBE1 on PC cell growth in vivo, we subcutaneously injected PC cells into the back of nude mice. Knockdown of GBE1 significantly reduced cell growth as a smaller tumor size was observed in GBE1 knockdown group (Fig. 3I, J, Fig. S2B), and the body weight change of nude mice was shown in Fig. 3K. To further confirm the role of GBE1 in PC cell tumorigenesis, we orthotopically injected PC cells into the pancreas of nude mice. As shown in Fig. 3L, M, GBE1 deficiency significantly decreased tumorigenesis of PC cells without no significant body weight change of nude mice between two groups. Our results indicated the oncogenic role of GBE1 in promoting tumorigenesis of PC cells in vivo. In summary, all the above data demonstrate the critical role of GBE1 in promoting cell growth and stemness-like properties of PC cells.

### WTAP and IGF2BP3 regulated GBE1 expression via m6A modification

**Fig. 4:**
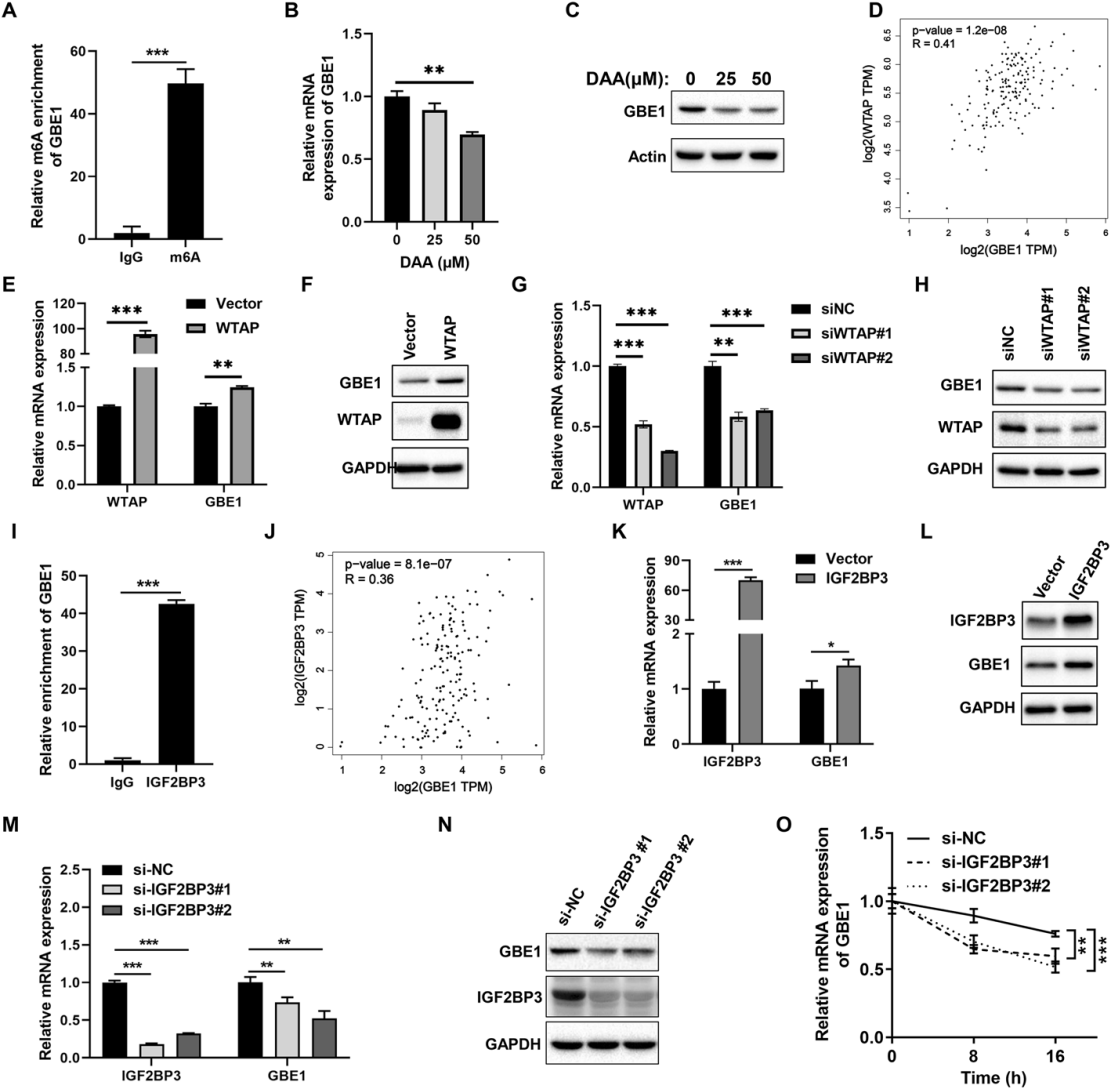
WTAP and IGF2BP3 mediated the m6A modification of GBE1.** A** m6A enrichment of GBE1 RNA by m6A RIP assay with m6A antibody (t test). mRNA (**B**) and protein (**C**) expression of GBE1 in BXPC3 cells after treatment with DAA (one way ANOVA test). **D** The positive correlation between WTAP and GBE1 mRNA expression as revealed in GEPIA platform. Overexpression of WTAP increased both RNA (**E**) and protein (**F**) expression of GBE1 (t test). Knockdown of WTAP by transfection with siRNAs reduced both RNA (**G**) and protein (**H**) expression of GBE1 (one way ANOVA test). **I** IGF2BP3 interacted with GBE1 RNA as revealed by RIP assay with IGF2BP3 antibody (*t* test). **J** The positive correlation between WTAP and GBE1 mRNA expression as revealed in GEPIA platform. Overexpression of IGF2BP3 increased both RNA (**K**) and protein (**L**) expression of GBE1 (t test). Knockdown of IGF2BP3 by transfection with siRNAs reduced both RNA (**M**) and protein (**N**) expression of GBE1 (one way ANOVA test). **O** Knockdown of IGF2BP3 by siRNA reduced GBE1 RNA stability in BXPC3 cells. BXPC3 cells were transfected with IGF2BP3 siRNAs and negative control siRNA. Twenty-four hours later, Actinomycin D was added to the transfected BXPC3 cells at a final concentration of 2ug/ml and total RNA was harvested at different time points. RT-qPCR analysis of GBE1 expression was performed (one way ANOVA test). *p < 0.05, **p < 0.01, ***p < 0.001

m6A modification is emerging as one of the most abundant RNA modifications involved in multiple processes of RNA post-transcription regulation [36]. To explore whether GBE1 underwent m6A modification, we analyzed m6A modification site of GBE1 mRNA sequence through SRAMP (http://www.cuilab.cn/sramp). As shown in Figure S3, several m6A modification sites with high confidence were uncovered. To further verify the m6A modification of GBE1, we performed RNA immunoprecipitation with m6A antibody (m6A-RIP). The result showed that there was a about 50-fold enrichment of GBE1 as compared to control IgG group (Fig. 4A). Then, we treated PC cells with 3-Deazaadenosine (DAA), a universal methylation inhibitor, thereby inhibiting m6A modification. As a results, DAA could reduce GBE1 expression in both RNA and protein levels (Fig. 4B, C, Fig. S1D). We also found that there was a positive correlation between methyltransferase WTAP and GBE1 (Fig. 4D). Furthermore, WTAP promoted GBE1 expression (Fig. 4E, F Fig. S1E), while knockdown of WTAP suppressed GBE1 expression (Fig. 4G, H, Fig. S1F). Thus, GBE1 was subjected to m6A modification, which was regulated by WTAP.

IGF2BP3 has been previously reported as a m6A reader in modulating RNA expression via m6A modification [37]. RNA immunoprecipitation (RIP) with IGF2BP3 antibody further confirmed that IGF2BP3 interacted with GBE1 mRNA, and 40-fold enrichment of GBE1 RNA was found when compared to control IgG group (Fig. 4I). Additionally, IGF2BP3 expression was positively correlated with GBE1 expression in PC via analysis in GEPIA platform (Fig. 4J). In order to demonstrate whether IGF2BP3 affected GBE1 expression, we overexpressed IGF2BP3 in BXPC3 cells, and then an upregulation of GBE1 was found in both RNA and protein levels (Fig. 4K, L,Fig. S1G). On the contrary, knockdown of IGF2BP3 by siRNAs suppressed GBE1 expression (Fig. 4M, N Fig. S1H). Furthermore, we analyzed the effected of IGF2BP3 on GBE1 RNA stability. IGF2BP3 siRNAs or negative control siRNAs were transfected into BXPC3 cells, then total RNA was harvested at different times after adding actinomycin D to the transfected BXPC3 cells. Loss of IGF2BP3 significantly enhanced RNA degradation of GBE1 (Fig. 4O). Therefore, IGF2BP3 bound to and stabilize GBE1 mRNA to facilitate GBE1 expression. In summary, WTAP and IGF2BP3 modulated GBE1 expression via m6A modification.

### GBE1 enhanced PC cell growth through upregulating c-Myc

**Fig. 5:**
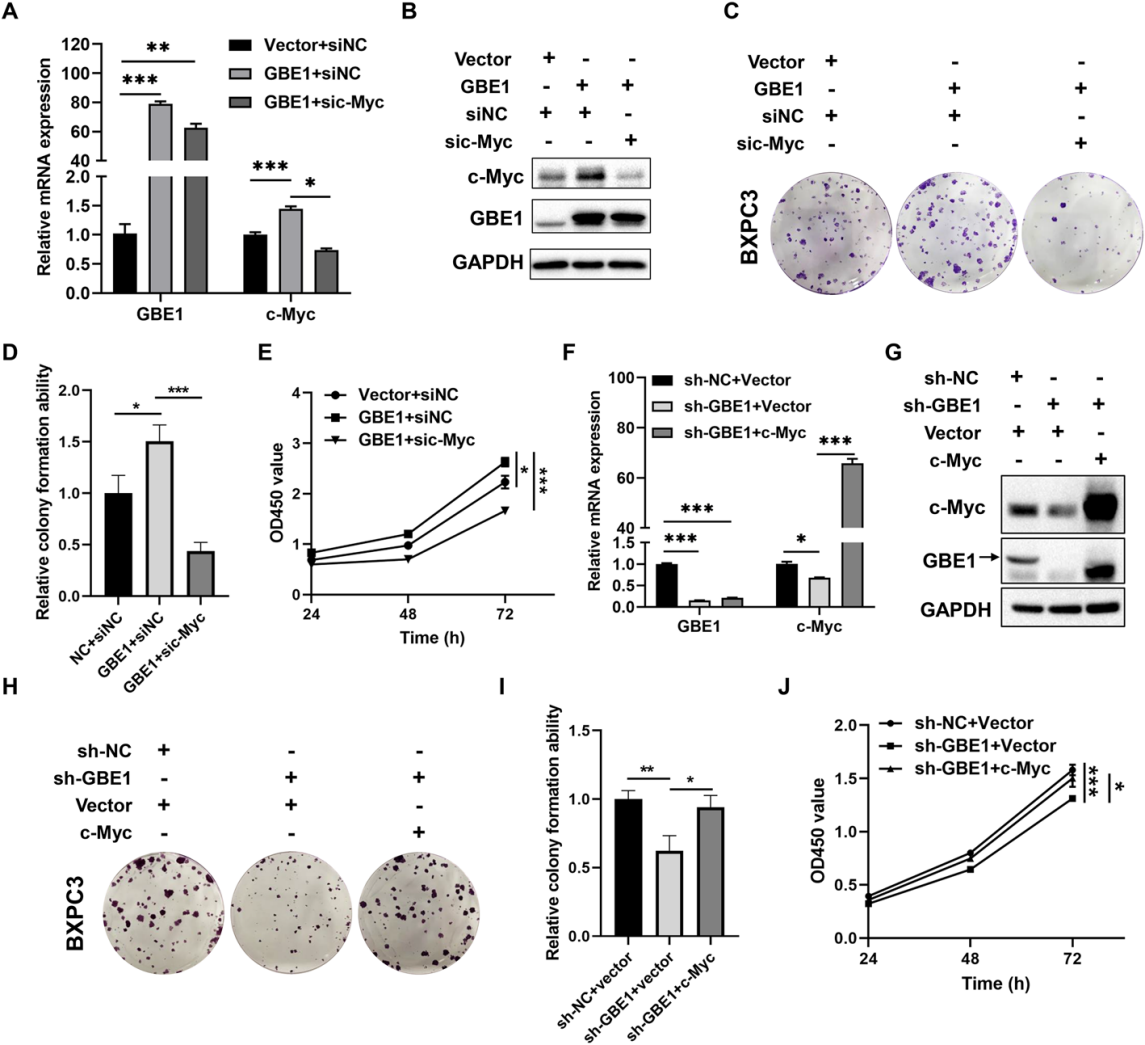
GBE1 enhanced PC cell proliferation by upregulating c-Myc. RT-qPCR (**A**) and Western blot (**B**) results of BXPC3 cells with knockdown of c-Myc after GBE1 overexpression (one way ANOVA test). **C** Colony formation assay indicated that c-Myc knockdown suppressed GBE1-induced cell proliferation. **D** Statistical analysis of colony formation results in C (one way ANOVA test). **E** CCK8 assay indicated that c-Myc knockdown suppressed GBE1-induced cell proliferation (one way ANOVA test). RT-qPCR (**F**) and Western blot (**G**) results of BXPC3 cells with overexpression of c-Myc after GBE1 knockdown (one way ANOVA test). **H** Colony formation assay indicated that c-Myc overexpression rescued GBE1 knockdown-induced cell proliferation suppression. **I** Statistical analysis of colony formation results in Figure H (one way ANOVA test). **J** CCK8 assay indicated that c-Myc rescued the cell proliferation inhibition induced by GBE1 knockdown (one way ANOVA test). *p < 0.05, **p < 0.01, ***p < 0.001

c-Myc is an oncogenic key transcription factor involved in gene expression, which is often upregulated in various human cancers and participate in regulating cell growth, chemoresistance and metabolism [30]. To clarify whether GBE1 functioned through regulating c-Myc expression, we detected c-Myc expression after overexpression or knockdown GBE1 respectively. As shown in Fig. 5A, B and Figure S1I, GBE1 overexpression remarkedly upregulated c-Myc expression. Functional rescue experiments showed that knockdown of c-Myc significantly attenuated GBE1-induced cell proliferation (Fig. 5C–E). On the contrary, knockdown of GBE1 decreased c-Myc expression (Fig. 5F, G Fig. S1J). Moreover, c-Myc overexpression rescued cell proliferation inhibition caused by GBE1 suppression (Fig. 5H–J). To further verify the regulation of GBE1 and c-Myc, IHC staining of GBE1, c-Myc and Ki67 in xenograft tumor tissues of this study was performed. As shown in Figure S4A, an upregulation of c-Myc level was observed in the tumor tissues upon GBE1 overexpression. And the proliferation marker-Ki67 was significantly enhanced by GBE1 overexpression (Fig. S4A). Moreover, decreased expression of c-Myc and Ki67 in GBE1 knockdown group was revealed in the tumor tissues of pancreatic subcutaneous and orthotopic xenograft model, which was consistent with our above findings (Fig. S4B-C). Therefore, GBE1 enhanced PC cell proliferation by upregulating c-Myc.

## Discussion

PC is one of the most aggressive human cancers accompanied by high risk of deaths. However, its etiology is still not well elucidated. A good knowledge of the molecular regulation in PC pathology and progression will be of great value to explore the targeted therapy for PC treatment. In this study, we found that GBE1 was highly expressed in both PC tissues and cell lines when compared with normal tissues and cell lines, and higher level of GBE1 predicted worse prognosis of PC patients, indicating that GBE1 might be a possible biomarker for PC.

Currently, little study about GBE1 has been reported in human cancers except LUAD. Studies have revealed that GBE1 enhanced LUAD cell growth, migration, stemness, glycolysis and anti-tumor immunity [28, 29], indicating GBE1 as a therapeutic target for LUAD. The potential function of GBE1 in pancreatic cancer progression have not been elucidated. Consistent with the oncogenic role of GBE1 in LUAD, we found that GBE1 promoted the cell growth and stemness-like properties of PC cells. Moreover, subcutaneous mouse model and orthotopic mouse model further demonstrated the role of GBE1 in facilitating PC tumor growth in vivo. These results demonstrated the oncogenic role of GBE1 in PC.

RNA m6A modification is one of the most abundant RNA modifications in RNA, which occurs in both coding RNAs and non-coding RNAs [4]. Currently, m6A modifications involved in the malignant progression of human cancers have been extensively reported [38, 39]. Increasing evidence have demonstrated that m6A level was significantly upregulated in both PC tissues and cell lines, when compared to normal tissues and cell line [14, 15, 17, 18, 40]. As reported, m6A modification modulated several RNA processes [4]. Our study revealed that m6A modification was present on GBE1 RNA. IGF2BPs, including IGF2BP1, IGF2BP2 and IGF2BP3, belongs to a conserved family of RNA-binding proteins to regulate RNA stability, translation and turnover of its targets such as IGF2, MYC, ACTIN and LIN28B [41–44]. Then, IGF2BPs was further identified as a family of m6A readers to promote the stability and translation of target RNAs through recognizing a conserved RRACH motif [10]. We previously reported that the oncofetal protein IGF2BP2, acted as a m6A reader, modulate LncDANCR expression in PC [13]. In this study, IGF2BP3 was identified as a m6A reader to enhance GBE1 mRNA stability and expression in PC cells, and WTAP, a m6A writer, also regulated GBE1 expression. All above findings indicated that WTAP/IGF2BP3 might regulate GBE1 expression in a m6A-dependent manner.

As reported, c-Myc, acting as popular transcription factor, was abnormally expressed in multiple human cancers and involved in regulating cell behaviors including cell proliferation, stemness, apoptosis, angiogenesis and metabolism through regulating its target genes, thereby facilitating tumor malignant progression [45–47]. Zhe et al. revealed that SHMT2 accelerating esophageal cancer development through enhancing c-Myc m6A modification and its expression [48]. LncRNA GLCC1 promoted colorectal cancer cell proliferation, glycolysis by enhancing c-Myc stabilization through suppressing c-Myc ubiquitination via binding to HSP90 [49]. Furthermore, METTL3 also triggered the malignant progression of Oral Squamous Cell Carcinoma by promoting m6A modification and stabilization of c-Myc [50]. At present, we found that GBE1 could positively regulated c-Myc expression. Importantly, functional rescue results showed that c-Myc can participate in the malignant behaviors of GBE1 in PC.

Additionally, there were limitations in our study. The m6A modification site in GBE1 mRNA that affected the binding of IGF2BP3 and whether there were other m6A regulators participated in the regulation of GBE1 m6A modification are both needed to be further demonstrated. Although we verify the binding between IGF2BP3 and GBE1 mRNA through RIP assay, the interaction between WTAP and GBE1 mRNA needs to be further explored. In current study, we showed that GBE1 could regulate c-Myc expression. However, the precise mechanisms by which GBE1 regulates c-Myc expression and activity are not fully elucidated, which can help us to better understanding of the oncogenic GBE1/c-Myc axis in PC progression. Additionally, it will be of interest to explore whether GBE1 involved in any other malignant behaviors of PC, since GBE1 are critical for glycogen accumulation and involved in anti-cancer immunity.

## Conclusions

In the present study, we firstly showed the higher expression of GEB1 in PC which was correlated with poor prognosis of PC patients. Mechanically, we revealed that WTAP and IGF2BP3 regulated GBE1 expression via m6A modification and also demonstrated that GBE1 facilitates PC cell proliferation, stemness-like properties and tumor growth. Moreover, c-Myc regulated by GBE1 was proved to be participate in the malignant behaviors of GBE1. Overall, our study uncovers the oncogenic GBE1/c-Myc axis in PC progression, and highlights the potential role of GBE1 in the prognosis and targeted therapy of PC.

### Supplementary Information

**Supplementary Material 1. **Fig. S1. Quantification of proteins expression. A Statistical analysis of protein expression in Fig. 1H (one way ANOVA). B Statistical analysis of protein expression in Fig. 2B (t test). C Statistical analysis of protein expression in Fig. 3B (*t* test). D Statistical analysis of protein expression in Fig. 4C (one way ANOVA). E Statistical analysis of protein expression in Fig. 4F (t test). F Statistical analysis of protein expression in Fig. 4H (two way ANOVA). G Statistical analysis of protein expression in Fig. 4L (*t* test). H Statistical analysis of protein expression in Fig. 4N (two way ANOVA). I Statistical analysis of protein expression in Fig. 5B (two way ANOVA). J Statistical analysis of protein expression in Fig. 5G (two way ANOVA). *p < 0.05, **p < 0.01, ***p < 0.001.

**Supplementary Material 2. **Fig. S2 Pictures of mice with tumors after overexpression or knockdown of GBE1. A Subcutaneous tumor-bearing mice in Fig. 2I before tumors were harvested. B Subcutaneous tumor-bearing mice in Fig. 3I before tumors were harvested.

**Supplementary Material 3. **Fig. S3 GBE1 m6A modification sites prediction results. GBE1 RNA sequence was subjected to the SRAMP (http://www.cuilab.cn/sramp) and then analyzed.

**Supplementary Material 4. **Figure S4. Immunohistochemical (IHC) staining of GBE1, c-Myc and Ki67. A IHC staining of xenograft tumor tissues in Fig. 2I. Statistical results were shown on the right (*t* test). B IHC staining of xenograft tumor tissues in Fig. 3I. Statistical results were shown on the right (*t* test). C IHC staining of xenograft tumor tissues in Fig. 3L. Statistical results were shown on the right (*t* test). Scale bar: 50 μm. *p < 0.05, **p < 0.01, ***p < 0.001.

## Data Availability

All data generated in current study was included in this published article. Data and materials are available from the corresponding author on reasonable request.

## Abbreviations

PC: Pancreatic cancer
GBE1: Glucan Branching Enzyme 1
CCK-8: Cell Counting Kit
OE: Overexpression
RIP: RNA immunoprecipitation
IARC: International Agency for Research on Cancer
m1A: N1-methyladenosine
m5C: 5-Methylcytosine
m7G: 7-Methylguanosine
ncRNAs: Non-coding RNAs
APBD: Adult polyglucosan body disease
GSD IV: Glycogen Storage Disease Type IV
LUAD: Lung adenocarcinoma
GEPIA: Gene Expression Profiling Interactive Analysis
SD: Standard deviation
OS: Overall survival
DSS: Disease specific survival
RFS: Relapse free survival
HPDE: Human pancreatic duct epithelial
CSCs: Cancer stem cells
m6A-RIP: RNA immunoprecipitation with m6A antibody
DAA: 3-Deazaadenosine
IHC: Immunohistochemistry
IOD: Integrated optical density
