# Supplementary figures and images for: WTAP/IGF2BP3-mediated GBE1 expression accelerates the proliferation and enhances stemness in pancreatic cancer cells via upregulating c-Myc
Source: Cell Mol Biol Lett. 2024 Jul 3;29:97. doi: 10.1186/s11658-024-00611-8 (PMC11223412; doi:10.1186/s11658-024-00611-8)

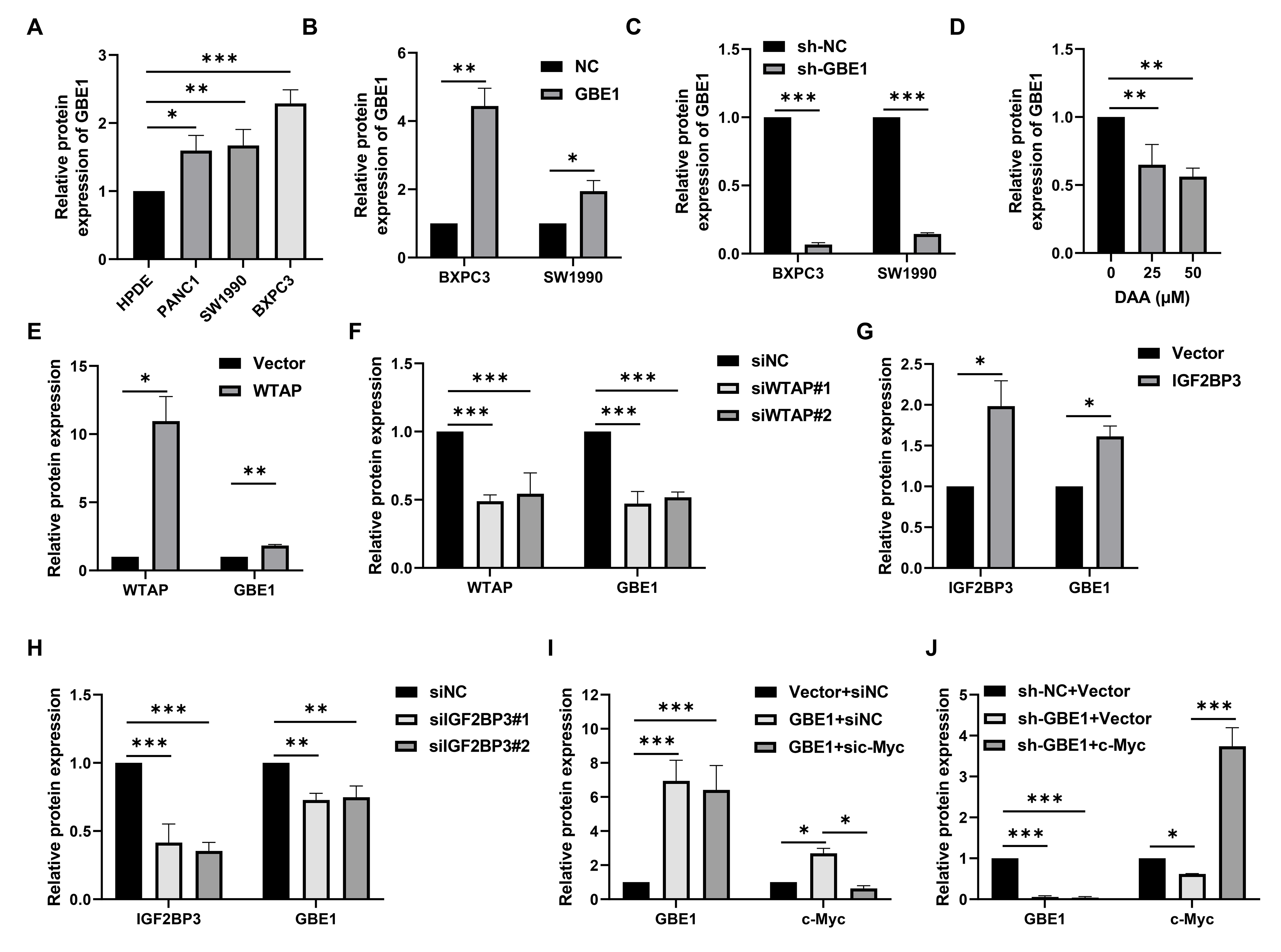

Supplement: Supplementary file 1 — Supplementary Material 1. Fig. S1. Quantification of proteins expression. A Statistical analysis of protein expression in Fig. 1H (one way ANOVA). B Statistical analysis of protein expression in Fig. 2B (t test). C Statistical analysis of protein expression in Fig. 3B (t test). D Statistical analysis of protein expression in Fig. 4C (one way ANOVA). E Statistical analysis of protein expression in Fig. 4F (t test). F Statistical analysis of protein expression in Fig. 4H (two way ANOVA). G Statistical analysis of protein expression in Fig. 4L (t test). H Statistical analysis of protein expression in Fig. 4N (two way ANOVA). I Statistical analysis of protein expression in Fig. 5B (two way ANOVA). J Statistical analysis of protein expression in Fig. 5G (two way ANOVA). *p < 0.05, **p < 0.01, ***p < 0.001. [file 11658_2024_611_MOESM1_ESM.tif]

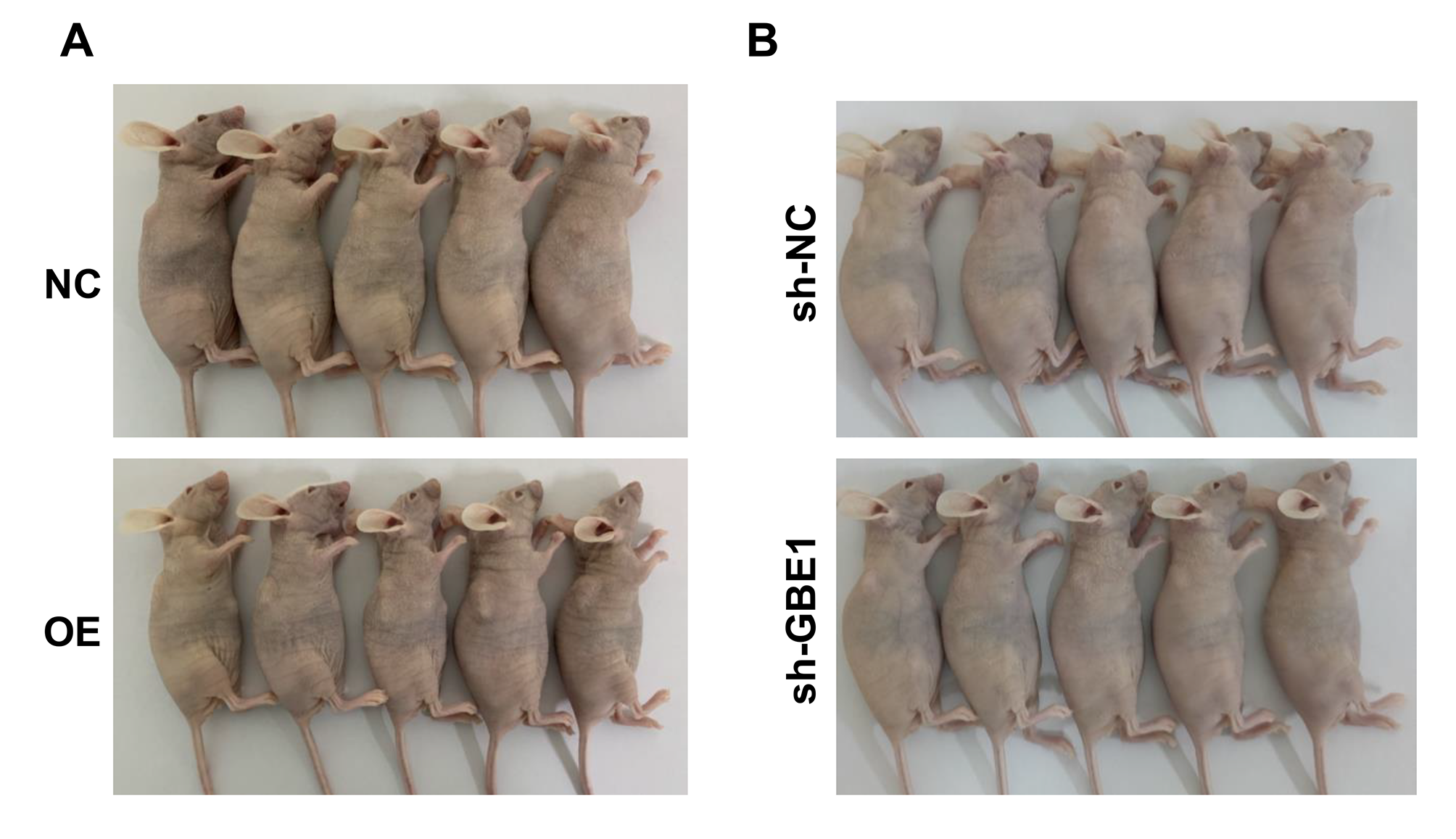

Supplement: Supplementary file 2 — Supplementary Material 2. Fig. S2 Pictures of mice with tumors after overexpression or knockdown of GBE1. A Subcutaneous tumor-bearing mice in Fig. 2I before tumors were harvested. B Subcutaneous tumor-bearing mice in Fig. 3I before tumors were harvested. [file 11658_2024_611_MOESM2_ESM.tif]

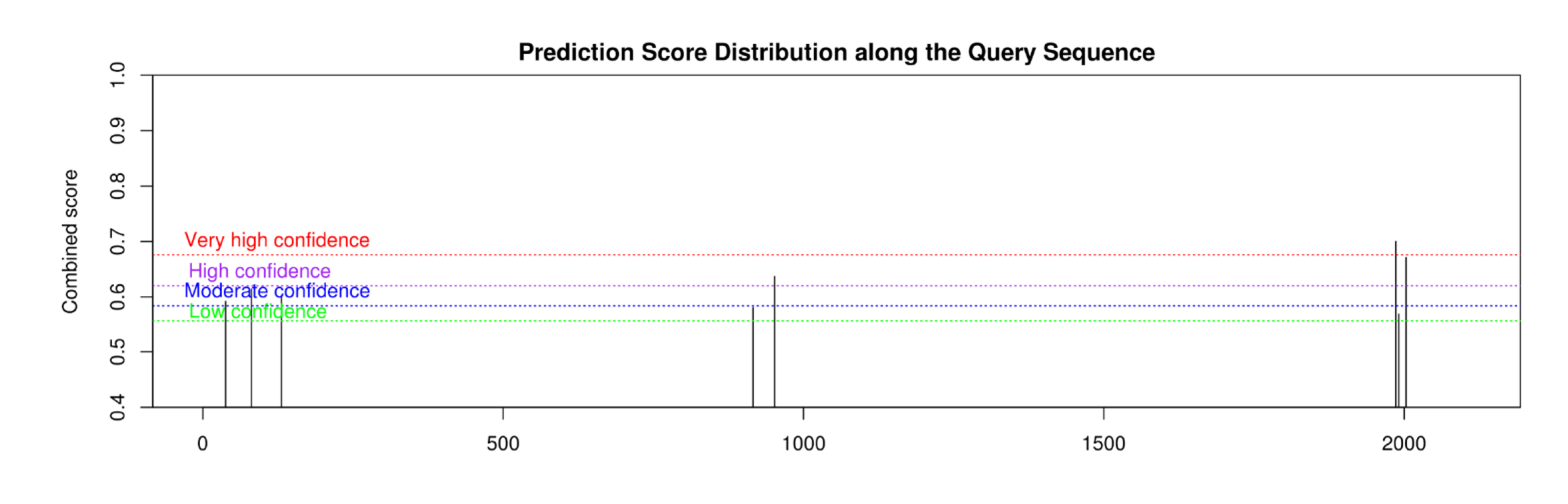

Supplement: Supplementary file 3 — Supplementary Material 3. Fig. S3 GBE1 m6A modification sites prediction results. GBE1 RNA sequence was subjected to the SRAMP (http://www.cuilab.cn/sramp) and then analyzed. [file 11658_2024_611_MOESM3_ESM.tif]

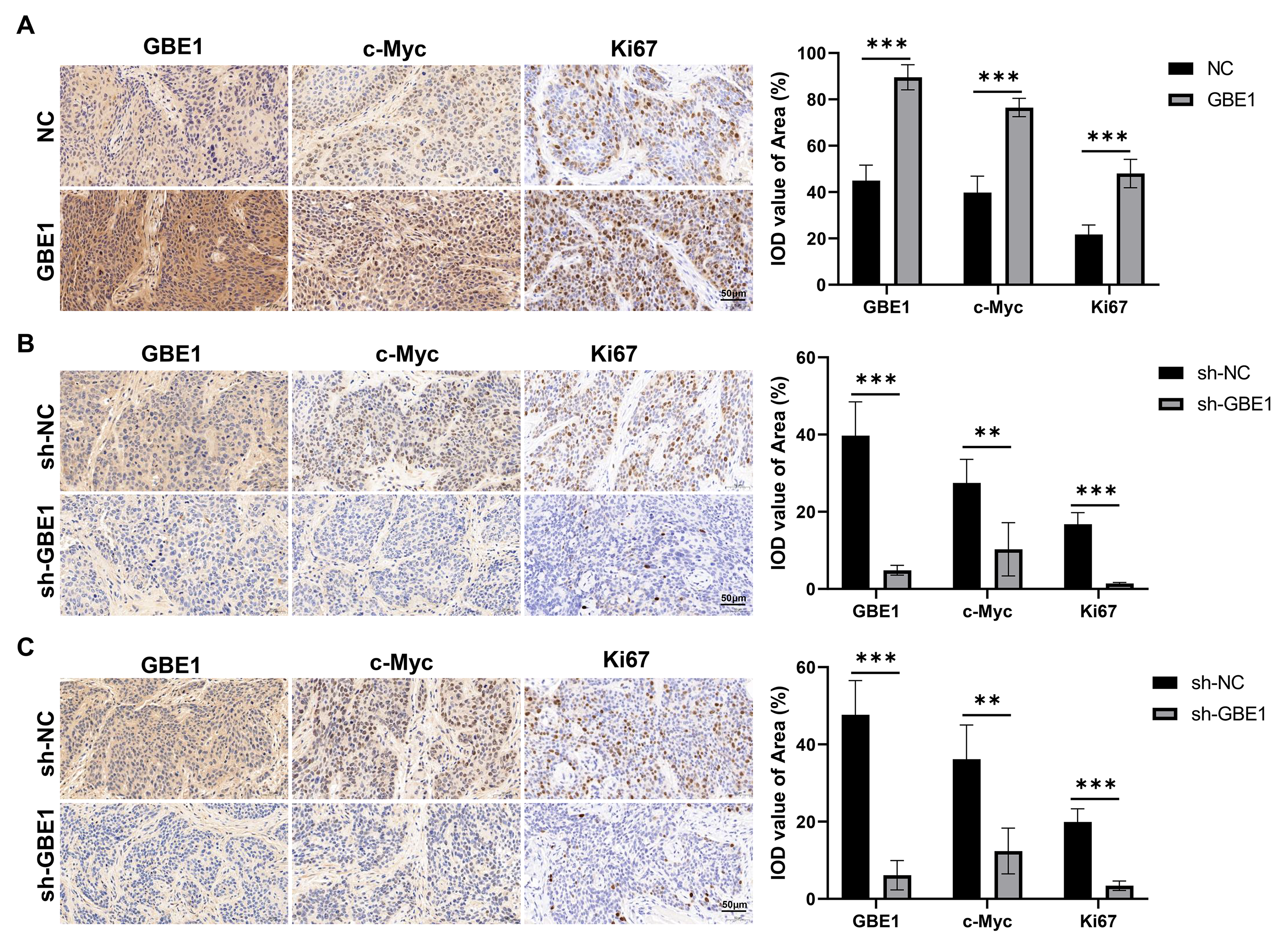

Supplement: Supplementary file 4 — Supplementary Material 4. Figure S4. Immunohistochemical (IHC) staining of GBE1, c-Myc and Ki67. A IHC staining of xenograft tumor tissues in Fig. 2I. Statistical results were shown on the right (t test). B IHC staining of xenograft tumor tissues in Fig. 3I. Statistical results were shown on the right (t test). C IHC staining of xenograft tumor tissues in Fig. 3L. Statistical results were shown on the right (t test). Scale bar: 50 μm. *p < 0.05, **p < 0.01, ***p < 0.001. [file 11658_2024_611_MOESM4_ESM.tif]
